# The neurotrophic effects of different human dental mesenchymal stem cells

**DOI:** 10.1038/s41598-017-12969-1

**Published:** 2017-10-03

**Authors:** Mallappa K. Kolar, Vinay N. Itte, Paul J. Kingham, Lev N. Novikov, Mikael Wiberg, Peyman Kelk

**Affiliations:** 1 Department of Integrative Medical Biology, Section for Anatomy, Umeå University, Umeå, Sweden; 2 Department of Surgical & Perioperative Sciences, Section for Hand and Plastic Surgery, Umeå University, Umeå, Sweden

## Abstract

The current gold standard treatment for peripheral nerve injury is nerve grafting but this has disadvantages such as donor site morbidity. New techniques focus on replacing these grafts with nerve conduits enhanced with growth factors and/or various cell types such as mesenchymal stem cells (MSCs). Dental-MSCs (D-MSCs) including stem cells obtained from apical papilla (SCAP), dental pulp stem cells (DPSC), and periodontal ligament stem cells (PDLSC) are potential sources of MSCs for nerve repair. Here we present the characterization of various D-MSCs from the same human donors for peripheral nerve regeneration. SCAP, DPSC and PDLSC expressed *BDNF*, *GDNF*, *NGF*, *NTF3*, *ANGPT1* and *VEGFA* growth factor transcripts. Conditioned media from D-MSCs enhanced neurite outgrowth in an *in vitro* assay. Application of neutralizing antibodies showed that brain derived neurotrophic factor plays an important mechanistic role by which the D-MSCs stimulate neurite outgrowth. SCAP, DPSC and PDLSC were used to treat a 10 mm nerve gap defect in a rat sciatic nerve injury model. All the stem cell types significantly enhanced axon regeneration after two weeks and showed neuroprotective effects on the dorsal root ganglia neurons. Overall the results suggested SCAP to be the optimal dental stem cell type for peripheral nerve repair.

## Introduction

Peripheral nerve injuries, especially those involving loss of tissue, are a serious clinical problem^1,2^. In these cases the current clinical gold standard treatment is the use of autologous nerve grafts^3^. However, nerve grafting necessitates the sacrifice of another nerve with resultant donor site morbidity^3^. Alternatives include natural or biosynthetic nerve conduits which may contain topographical guidance cues and integrated growth factors to promote axonal regeneration^4–6^. Despite much research, the artificial conduits remain largely inferior to the autologous nerve grafts. Boosting the growth permissive environment of the conduits can be achieved with transplantation of autologous Schwann cells (SCs), but these are available in limited quantities and harvest is associated with donor site morbidity.

Adult mesenchymal stem cells (MSCs) can promote regeneration after peripheral nerve injury^7,8^. Adipose tissue derived (ASCs) and bone marrow derived cells (BM-MSCs) have been shown to produce neurotrophic factors, which can enhance neurite outgrowth and provide neuroprotection *in vivo*, and serve as alternative to SCs for nerve repair^7,9,10^. Dental MSCs (D-MSCs) comprise stem cells from apical papilla (SCAP), dental pulp stem cells (DPSC), periodontal ligament stem cells (PDLSC), periodontal ligament of deciduous teeth stem cells, dental follicle progenitor cells and stem cells from human exfoliated deciduous (SHED)^11^. Most studies suggest that D-MSCs share the same characteristic mesenchymal stem cell surface markers as ASCs and BM-MSCs, however differences in proliferation and differentiation potential have been noted. DPSC proliferate faster than BM-MSCs and generate more colony forming units^12^. Furthermore, differentiation assays showed that the osteogenic potential of DPSC was greater than BM-MSCs whereas the opposite was the case for adipogenic differentiation^12^. In another study, micro-computed tomography also demonstrated that osteogenic differentiation was significantly greater in DPSC compared with BM-MSCs and ASCs, although the differentiated dental cells did not express distinct nodular morphology^13^. Comparisons of stem cell niche, homing, and immunoregulatory properties of D-MSCs versus BM-MSCs have also been described^14^. Despite the potential advantages of using dental tissue-derived MSCs for bone regeneration the ability to extract enough cells for therapeutic application is a limiting factor. Development of stem cell banks to store expanded numbers of D-MSCs obtained from deciduous and extracted permanent teeth obtained at different stages of life will enable the provision of cells when needed.

In contrast to the well-developed field of D-MSCs for bone tissue engineering, until recently, there have been few studies of their use for nerve regeneration. Since D-MSCs are derivatives of the neural crest^14^, they may have advantages for treatment of nerve injury and neurodegenerative diseases, as compared to other stem cell types. A few studies have shown the effects of DPSC^15^, SHED^16^ and PDLSC^17^ on regeneration after nerve injury. The positive benefits are likely, in part, to be mediated by the stem cells secretome^18^. For instance, DPSC derived Schwann-like cells have been shown to express various neurotrophic factors^15^. Human SHED have been induced to differentiate into neuron-like cells with increased expression of neurotrophin-3 (NT3) and neurotrophin-4/5^19^. Furthermore, brain derived neurotrophic factor (BDNF) secretion from SCAP plays a key role in neurite outgrowth *in vitro* ^20^. As far as we are aware, there are no direct comparisons of the effects of different populations of D-MSCs obtained from the same individuals on peripheral nerve regeneration. Therefore in this study we compared different populations of D-MSCs isolated from same human donors and examined their neurotrophic properties and effects on regeneration after sciatic nerve injury and repair in adult rats.

## Results

### Stem cell characterisation

**Figure 1:**
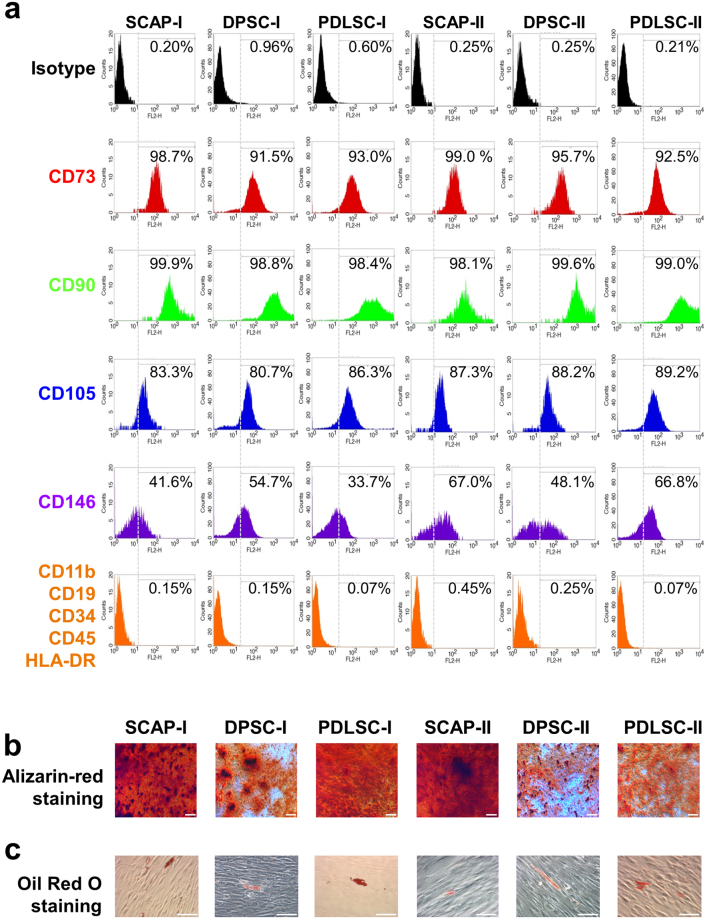
Human D-MSCs characterization, osteogenic, and adipogenic differentiation. Flow cytometry analysis of human SCAP, DPSC and PDLSC from two donors (marked as I and II) at passage 2, were positive for expression of CD73, CD90 and CD105 and lacked the expression of the negative markers (HLA-DR, CD45, CD34, CD19 and CD11b) **(a)**. The expression profiles of the MSCs markers were inter- and intra-individually similar among the various D-MSCs (**a**). Differentiation along osteogenic lineage (Alizarin red) and adipogenic lineage (Oil Red O) is shown in (**b**) and (**c**), respectively. Scale bar: 100 μm.

Adherent D-MSCs cultures from early passages were analysed using flow cytometry. All three populations showed high expression of mesenchymal stem cell markers (CD73, CD90, CD105, CD146) and negligible expression of the negative control markers of CD11b, CD45, HLA-DR, CD19 and CD 34 (Fig. 1a). After four weeks of differentiation, each type of D-MSCs differentiated along the osteogenic and adipogenic lineages as shown with Alizarin red staining (Fig. 1b) and Oil Red O staining (Fig. 1c) respectively.

### Growth factor expression of D-MSCs and effects on *in vitro* neurite outgrowth

**Figure 2:**
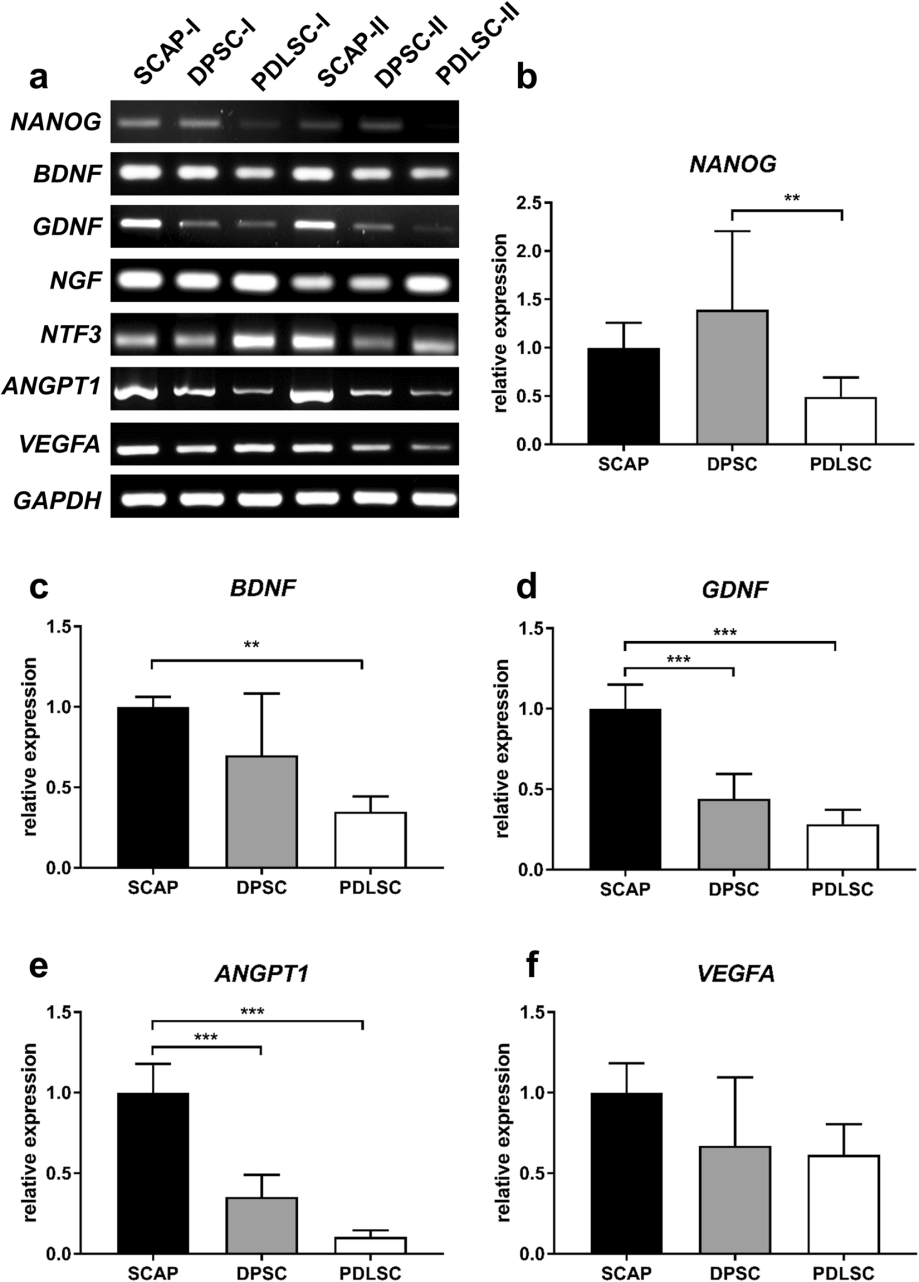
Neurotrophic and angiogenic factor gene expression of D-MSCs. RT-PCR analysis of various unstimulated D-MSCs at passage 2, showed a variability of gene expression in unstimulated D-MSCs. *GAPDH* is used as a house-keeping gene. For original and uncropped blots, see Supplementary Figure 1. Quantitative-PCR analyses for *NANOG* (**b**), *BDNF* (**c**), *GDNF* (**d**), *ANGPT1* (**e**) and *VEGFA* (**f**) with *GAPDH, ACTB and RPL13A* used for normalisation and relative expressions set to value 1 for the SCAP group. P < 0.01 is indicated by ** and P < 0.001 is indicated by *** (n = 2 patients and each condition was performed with 4 technical replicates).

Semi-quantitative RT-PCR was first used to confirm expression of an embryonic stem cell marker gene *(NANOG)* and assess the neurotrophic and angiogenic profiles of each D-MSCs population from two patients. The levels of *BDNF, GDNF, ANGPT1* and *VEGFA* were highest in SCAP followed by DPSC and PDLSC in both patients (Fig. 2a). There was variation in *NTF-3* expression levels. *NGF* expression was similar between D-MSCs in patient I but PDLSC were found to have highest expression in patient II. Quantitative RT-PCR confirmed that there were significantly higher levels of *BDNF*, *GDNF* and *ANGPT1* in the SCAP versus the other D-MSCs (Fig. 2c–e).

**Figure 3:**
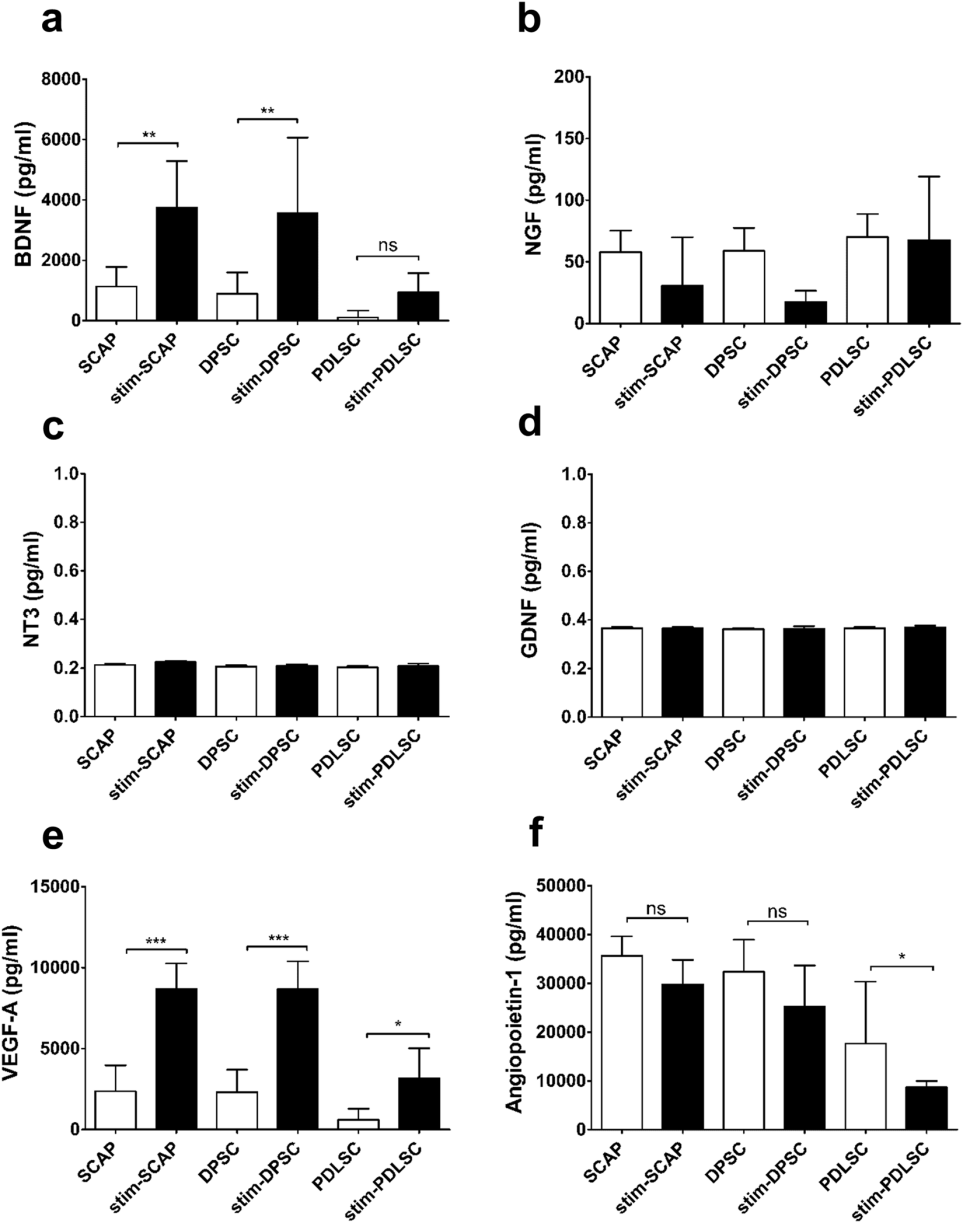
Quantification of secreted protein in supernatants from unstimulated D-MSCs (cultured in regular MSCs medium) and stimulated D-MSCs (cultured for 14 days in MSCs-medium supplemented with neuregulin1-β1, basic fibroblast growth factor, platelet derived growth factor and forskolin). ELISA analysis of stimulated D-MSCs showed significantly elevated protein levels of BDNF **(a)** and VEGF-A **(e)** in SCAP and DPSC groups compared with corresponding unstimulated controls. In contrast, the stimulation protocol did not induce PDLSC to secrete significantly higher levels of BDNF **(a)**. In addition the stimulation of D-MSCs did not lead to elevated secretion of NGF **(b)**, NT3 (**c**), GDNF **(d)**, or angiopoietin-1 **(f)**. All levels were normalized to 10^6^ cells per group (pg/ml = pg/10^6^ cells). Mean values +/− SD are indicated, n = 6 independent experiments from 2 different donors for each D-MSCs group. P < 0.05 is indicated by *, P < 0.01 is indicated by ** and P < 0.001 is indicated by ***.

The levels of growth factors secreted from D-MSCs were also measured by ELISA. Using a previously published protocol^7^, we stimulated the D-MSCs with a mixture of growth factors and this produced a significant increase in the production of BDNF in SCAP and DPSC but not in PDLSC (Fig. 3a). No changes in the production of NGF, NT-3, or GDNF were observed in any of the stimulated D-MSCs (Fig. 3b–d). All types of D-MSCs significantly increased VEGF-A production following the stimulation protocol (Fig. 3e), while the same treatment decreased production of angiopoietin-1 (Fig. 3f).

**Figure 4:**
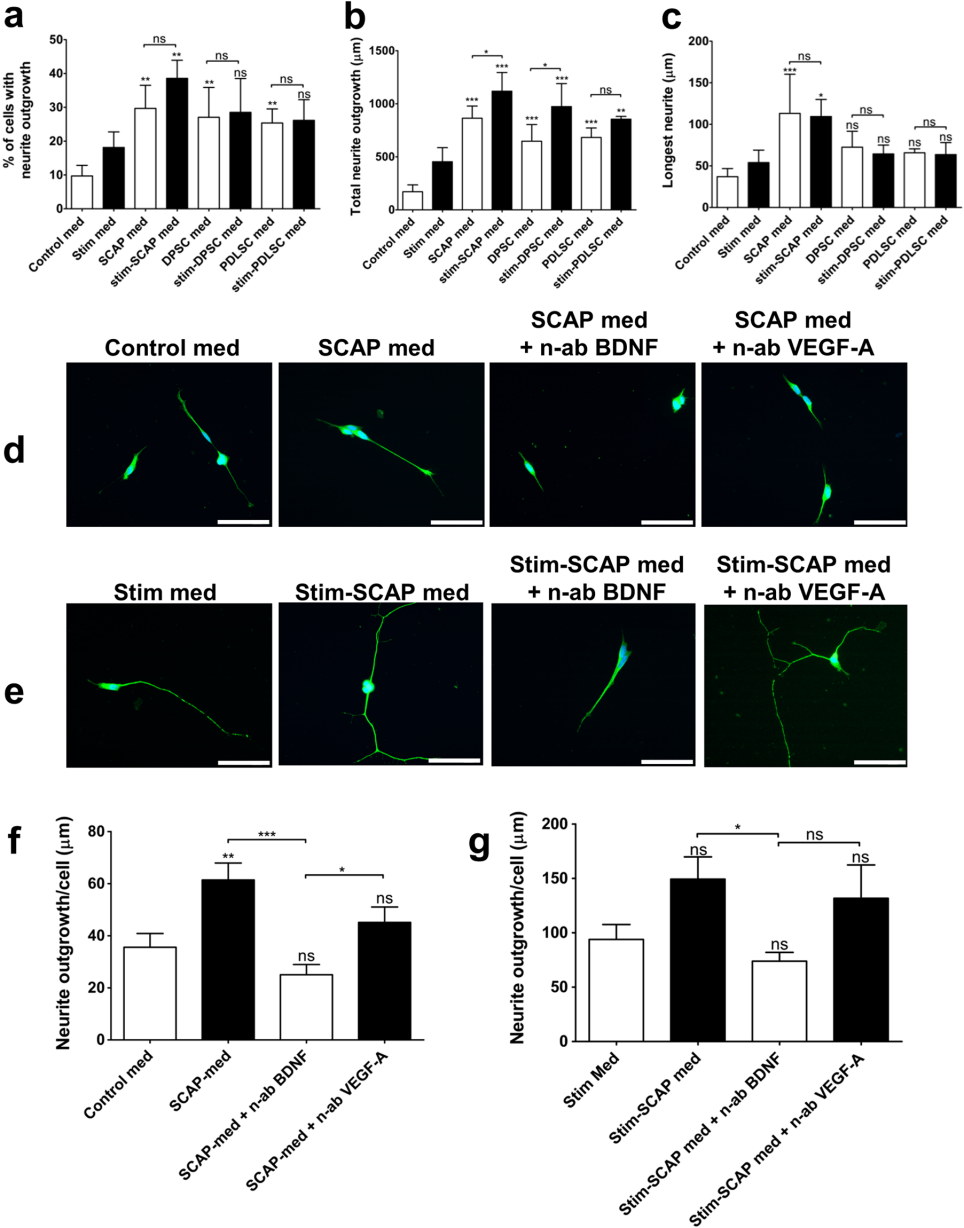
Neurite outgrowth of SHSY-5Y neuronal cells exposed to various conditioned media from D-MSCs. SHSY-5Y cells were retinoic acid-differentiated for 48 h (**a**–**c**) or 72 h (**d**–**g**) prior to exposure to conditioned media. Thereafter, retinoic acid-differentiated SHSY-5Y cells were exposed to control MSCs-medium (=control med), unstimulated D-MSCs conditioned medium (=SCAP, DPSC, or PDLSC med), stimulation medium alone (containing neuregulin1-β1, basic fibroblast growth factor, platelet derived growth factor and forskolin, =stim med), or stimulated D-MSCs conditioned medium (D-MSCs cultured for 14 days in MSCs-medium supplemented with neuregulin1-β1, basic fibroblast growth factor, platelet derived growth factor and forskolin, =stim-SCAP, stim-DPSC, or stim-PDLSC med). Exposure to various control or conditioned media was for additional 48 h (**a**–**c**) or 72 h (**d**–**g**). Quantitative analyses (n = 400 cells/group) of βIII-tubulin immunostained SHSY-5Y neurons exposed to various media are shown for the percentage of cells with neurite outgrowth **(a)**, total neurite outgrowth **(b)**, and longest neurite length **(c)**. SCAP-groups were further analysed for possible effects of secreted factors on neurite outgrowth, by utilizing neutralizing antibodies (n-ab) against BDNF or VEGF-A (**d**–**g**). Representative βIII-tubulin immunostaining (400X magnification) of SHSY-5Y neurons showed decrease of neurite outgrowth upon blockage of BDNF, but not for VEGF-A (**e–g**). Quantitative analyses of SHSY-5Y neurites/cell (n = 38–41 cells/group) from (**d**) are shown in (**f**), and from (**e**) are presented in (**g**). Mean values with error bars showing SD **(a**–**c)** and SEM (**f**,**g**). Statistical significance are indicated with *P < 0.05; **P < 0.01; ***P < 0.001 or if not significant (ns). Scale bar: 50 μm.

To examine the biological activity of the conditioned media, a neurite outgrowth assay was used. Differentiated human neuroblastoma SH-SY5Y cells were incubated in media taken from the unstimulated or stimulated D-MSCs (Fig. 4a–c). Quantification of the neurite outgrowth showed that unstimulated and stimulated D-MSCs increased both the percentage of cells producing neurites (Fig. 4a) and the total neurite outgrowth length (Fig. 4b), when compared with the respective medium-only controls. However, the length of the longest neurite per neuron was only significantly increased in the presence of SCAP-conditioned media (Fig. 4c). Further analysis of the SCAP groups revealed that neutralising secreted BDNF inhibited the neurite outgrowth evoked by unstimulated SCAP (Fig. 4d and f) and stimulated SCAP conditioned media (Fig. 4e and g). Neutralization of VEGF-A did not affect neurite outgrowth (Fig. 4d–g).

### D-MSCs enhance axon regeneration *in vivo*

**Figure 5:**
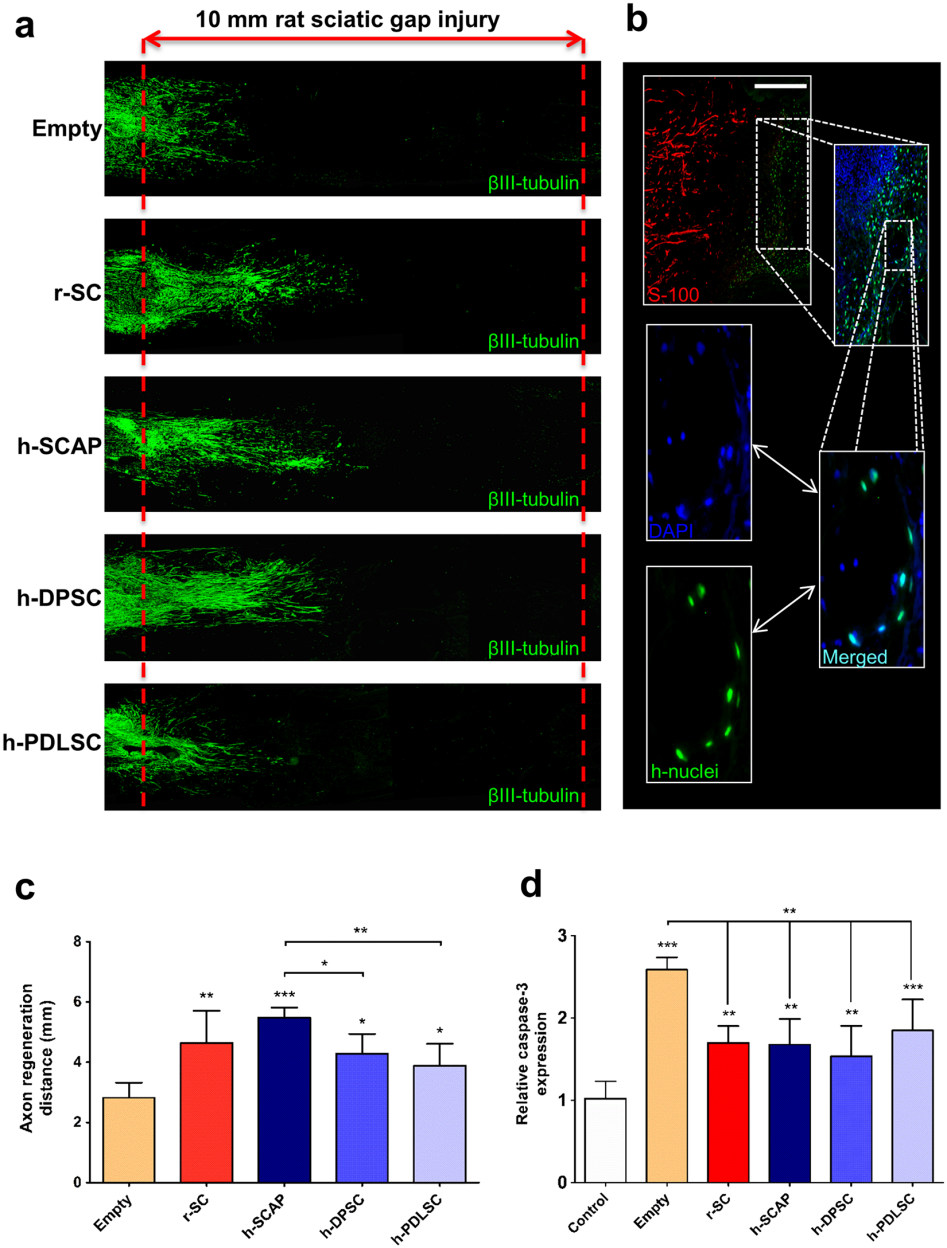
The effect of transplanted human D-MSCs on nerve regeneration *in vivo*. βIII-tubulin staining of longitudinal sections through the nerve conduit showing regeneration from the proximal stump into the 10 mm gap after 2 weeks. Representative stainings are shown for empty conduits (Empty: n = 5), rat Schwann cells (r-SC: n = 5), human-SCAP (h-SCAP: n = 4 donor I, n = 4 donor II), human DPSC (h-DPSC: n = 3 donor I, n = 3 donor II), or human PDLSC (h-PDLSC: n = 3 donor I, n = 3 donor II) **(a)**. The presence of human nuclei-specific antigen (h-nuclei; green) showed transplanted cells (shown for donor II) in the conduit after 2 weeks. Note that there are no co-localizations with S100 antigen (red) **(b)**. The distance of the axon regeneration in the conduit (**c**) and Caspase-3 expression in DRG **(d)**. Axon regeneration was measured in control empty conduits (Empty: n = 5), conduits with rat SC (n = 5) and conduits with unstimulated D-MSCs (h-SCAP: n = 4 donor I, n = 4 donor II; h-DPSC: n = 3 donor I, n = 3 donor II; or h-PDLSC: n = 3 donor I, n = 3 donor II) **(c)**. Significance levels of *P < 0.05; **P < 0.01; ***P < 0.001 are indicated. Caspase-3 gene expression in L4-L6 DRG from rats with no injury (Control), or after nerve injury and repair (Empty, rSC, h-SCAP, h-DPSC, or h-PDLSC). Relative expression levels are shown with regard to control samples (value = 1) **(d)**. Connecting lines show relative significance; *P < 0.05, **P < 0.01, and ***P < 0.001, n.s., not significantly different. n = 5 from pooled animal RNA samples (5–8 animals per group). Mean values + /−SD are indicated. Scale bar: 250 μm.

At 2 weeks after rat sciatic nerve injury, immunohistochemistry demonstrated the greatest distance of regeneration occurred in animals treated with conduits containing rat Schwann cells (rSC) or with unstimulated SCAP (Fig. 5a and c). Although less than the SCAP and rSC groups, the increase in the distance of regeneration in conduits seeded with DPSC and PDLSC was statistically significant compared to the empty conduit group (Fig. 5a and c). Human nuclei antibody staining demonstrated that D-MSC could be found *in vivo* after the 2-week period. The D-MSCs were found in close proximity to the SC at the proximal regeneration front (Fig. 5b). The results also demonstrated that transplanted D-MSCs did not express the glial marker S-100 (Fig. 5b). The antibodies to human nuclei did not label the rat cells (see Supplementary Fig. S3). *In vivo* expression of BDNF was located in the vicinity of the transplanted human cells (see Supplementary Fig. S4) suggesting that the cells maintain the expression of this growth factor which is produced *in vitro* (see Supplementary Fig. S4). Peripheral nerve injury and repair with empty conduits showed a 3-fold increase in caspase-3 expression levels (Fig. 5d). Conduits seeded with rSC or D-MSCs significantly reduced caspase-3 expression compared with the empty conduit group (Fig. 5d).

## Discussion

The extraction of wisdom teeth is one of the most common procedures performed in oral and maxillofacial surgery^21^. As a potential stem cell source, teeth are easily accessible, harbour a number of different cell populations and yield a high number of rapidly growing but controlled, multipotent stem cells. In this study we compared 3 different populations of human D-MSCs from two donors with matched tooth developmental stage, for their expression of growth factors and effects on peripheral nerve regeneration. We found that all types of D-MSCs produce various levels of neurotrophic and angiogenic factors and promote nerve regeneration. However, one of the major limitations of our study is the small sample size due to exclusion of teeth that were not matched regarding their developmental stage and from which we could not isolate all the forms of D-MSCs (in this case SCAP, DPSC, and PDLSC). Nevertheless, we believe that teeth isolated from donors I and II represent healthy teeth and are considered as representative healthy clinical samples. Ideally, further matched donors with teeth at the same developmental stage as for donor I and II should be collected to confirm our results. A main challenge due to ethical limitations and the clinical situation, is to find matched donors with teeth at similar developmental stage that can be used as research material.

Recent studies in mice have shown that D-MSCs can develop from glial cells^22^ or neural crest cells^23,24^. A number of different D-MSCs populations have been cultured from the teeth and in this study, we isolated SCAP, DPSC and PDLSC populations from two matched human donors and showed they exhibited typical properties of MSCs^25–27^. Of the stem cell markers tested, we found that expression of CD146 was most variable in the cells at both the intra and inter-donor level. Previous studies have shown a correlation between pluripotency genes, CD146 and the potential for osteogenic differentiation^28^; however, this was not evident from our experiments. The SCAP cultures clearly showed more intense calcified nodule formation than the other two populations of dental cells. DPSCs have been shown to exhibit inferior osteogenic differentiation compared with other types of oral cavity stem cells^29^, but also display a different pattern of mineralisation in comparison with non-dental derived MSCs^13^.

DPSC have been the most commonly used type of dental stem cells in models of nerve injury^15,25,30^, with a few studies investigating other types of D-MSCs^16,17,20,31^. In Sakai *et al*., 2012 only two types of D-MSCs (SHED and DPSC) were used and they were not from the same individuals^25^. Using matched MSC donors is very important considering inter-individual variability. Our study demonstrated for the first time, that SCAP, DPSC, and PDLSC from matched donors, can promote axon regeneration when delivered within biosynthetic conduits. The results also showed that D-MSCs had similar effects on nerve regeneration as previously reported for ASCs^7^. These findings are in line with previous report that DPSC-enhance neuroprotection and neurogenesis on retinal ganglion cells *in vitro* ^32^. We found that transplantation of the three different D-MSCs populations and rat Schwann cells significantly decreased the pro-apoptotic gene, caspase-3, expression in the DRG. Similar results have been reported previously using DPSC^33^ and ASCs^7,34^. The findings that transplanted D-MSCs do not express the glial marker S-100 are also consistent with previous observations^33^.

In our initial characterisation of the various D-MSCs we found that *BDNF*, *GDNF* and *ANGPT1* genes were expressed at higher levels in the SCAP versus DPSC and PDLSC. However, of these molecules only BDNF was shown to have correspondingly higher levels of protein secretion from the SCAP. Furthermore, we were unable to detect significant levels of secretion of other neurotrophic factors for which we detected mRNA transcripts. This can be explained by the fact that compared with transcription, post-transcriptional modulators e.g. microRNAs and translational or post-translational events play an important role in determining overall accumulation and release of the mature proteins. Furthermore, given the sensitivity of the PCR technique it is conceivable that we can detect transcripts expressed in a small sub-population of the D-MSCs cultures. Indeed, even for BDNF, we found that not all the cells in SCAP cultures had detectable levels of protein (see Supplementary Fig. S4). Crigler *et al*. showed similar sub-populations of BDNF expressing stem cells in cultures of human bone marrow derived stem cells^35^.

Boosting trophic factor production in MSCs has been shown to have a major impact on regeneration^36–39^. We and other research groups have developed protocols to stimulate MSCs with growth factors and/or differentiate them towards Schwann cell-like phenotypes^7,10,40,41^. This treatment leads to enhanced production of various growth factors and improves nerve regeneration. By using the protocol, we found that stimulation of the D-MSCs led to a significant increase in secretion of BDNF and VEGF-A proteins. Conditioned media from all the D-MSCs enhanced neurite outgrowth in an *in vitro* model and the stimulation process significantly further enhanced the total neurite outgrowth in the SCAP and DPSC cultures. Only SCAP and stimulated-SCAP-conditioned media significantly enhanced the length of the longest neurites in our *in vitro* model. This could, at least in part, be explained by the action of BDNF, since it acts synergistically with retinoic acid to mature differentiating SHSY-5Y neurons^42^. Furthermore, we could entirely block SCAP-conditioned-media-induced neurite outgrowth by utilizing neutralizing antibodies to BDNF in our *in vitro* model. This observation partially elucidates the important role of secreted BDNF involved in our results and is in line with recently reported findings^20^. Since stimulated DPSC produced the same levels of BDNF as found in cultures of stimulated SCAP, but had no significant effect on longest neurite length, it is likely that other factors produced by the DPSC in some way impede axonal elongation/maturation and instead enhance sprouting (at least at the time point recorded in this study).

Although the stimulation process did potentiate the D-MSCs effect on neurite outgrowth, the effect was rather limited, so we chose to use the unstimulated cells for testing *in vivo*. The unstimulated D-MSCs induced significant regeneration compared to cell-free nerve conduits. Consistent with the *in vitro* results, the SCAP group produced the longest distance of axonal regeneration. This might, in part, be due to continued production of BDNF, since we noted immunostaining for BDNF in the vicinity of transplanted cells, even after 2 weeks *in vivo* (see Supplementary Fig. S4). Nevertheless, it is possible that effects on axonal regeneration could be also due to well-known immunomodulatory effects and production of various extracellular matrix molecules by MSCs^14,43,44^. The transplanted D-MSC are also likely to interact with the endogenous Schwann cells which proliferate after nerve injury. Schwann cells and DPSC act synergistically *in vitro* to produce high levels of BDNF and GDNF and when both cell types are transplanted together in nerve conduits there are improvements in histological and electrophysiological outcomes compared with animals treated with each cell type alone^45^. DPSC have also been shown to reduce apoptosis and increase proliferation in resident Schwann cells following nerve injury^46^. The growth factor-rich D-MSCs secretome could also influence the survival of the primary sensory neurons which also die because of nerve injury. For example, the DPSC secretome has been shown to stimulate the endogenous survival factor Bcl-2 and decrease the apoptotic regulator Bax^18^.

In conclusion, in this study we have shown that human SCAP, DPSC and PDLSC from the same donors can provide an alternative to Schwann cells to support regeneration after peripheral nerve injury and repair. Overall the results suggest that SCAP might have the greatest benefits.

## Methods

### Dental MSCs isolation

All procedures were approved by the local ethics committee for Clinical Research in Umeå University (No. 03-425) and all methods were performed in accordance with the relevant guidelines and regulations of the local ethics committee for Clinical Research in Umeå University. Informed consent was obtained from all subjects and/or their legal guardians. Seven donors with impacted premolars or third molars that were aimed to be surgically removed due to planned orthodontic treatment or as pre-treatment before orthognathic surgery were selected for this study. The selected teeth with at least 30% root formations were surgically removed from seven donors (age range 12–25 years) at the Maxillofacial Surgery Section at the University Hospital, Umeå. Of the seven donors, only two donors (n = 2) had impacted teeth with dental follicle that only covered the “crown-part” of the teeth. In the surgically removed teeth from these two donors, the various dental-MSCs could easily be distinguished: (Donor I: two retained maxillary second premolars from a 12-year old female; Donor II: two retained mandibular third molars from an 18-year old female). The teeth from the selected two donors were at similar tooth developmental stage (with approximately 70% of root-formation completed). Human SCAP, DPSC and PDLSC (for schematic illustration of collection sites, see Supplementary Fig. S1) were isolated from the teeth by mincing followed by digestion in 3 mg/ml collagenase type I (Worthington Biochemicals Corp.) and 4 mg/ml dispase II (Roche Diagnostic/Boehringer Mannheim Corp.) for 60 minutes at 37 °C and 5% CO_2_. Single cell suspensions of SCAP, DPSC and PDLSC were obtained by passing the cells through a 70 µm strainer (Falcon, BD Labware). Cells were seeded at 10^4^ cells/25 cm^2^ flask (Costar), and cultured in Minimum Essential Medium-α (MEM-α with GlutaMax; Invitrogen, Carlsbad, USA) supplemented with 15% (v/v) foetal bovine serum (FBS; Invitrogen), 100 mM L-ascorbic acid 2-phosphate (WAKO, Tokyo, Japan), 2 mM L-glutamine (Biosource/Invitrogen) and 1% (v/v) penicillin/streptomycin solution (PAA) at 37 °C in 5% CO_2_. On reaching ∼90% confluence, cells were passaged to new 75 cm^2^ culture flasks using trypsin/EDTA solution (Invitrogen) and plated at a density of 5,000 cells/cm^2^. D-MSCs were successfully expanded from these two donors through to passage 4, as previously described^47,48^. Cells originating from different teeth, but from same type of D-MSC and donor were pooled (for instance: DPSC from premolar 1 and premolar 2 from donor I) after passage 0 (initial culture). Cells between passages 1 and 4 were used in the following *in vitro* and *in vivo* experiments; comparisons were made on cells at matching passage numbers.

### Flow cytometry

D-MSCs at early passage (p1-2) were collected and tested for positive MSCs-associated surface markers (CD73, CD90, CD105, and CD146) and negative markers (CD11b, CD19, CD34, CD 45 and HLA-DR), to define the cells as MSCs^14^ according to the manufacturer’s protocol (BD Bioscience). All antibodies used for FACS analysis were PE-conjugated. Optimal concentrations of antibodies were calculated (1:25 for CD73, 1:33 for CD90, 1:25 for CD 105, and 1:25 for CD146) and 5000 cells for each analysis were chosen. As negative control, a corresponding isotype control was used for each sample (mouse IgG1, κ). Data was acquired using FACSCalibur (BD Bioscience) as previously described^48^.

### Osteogenic & adipogenic differentiation

The multi-potency of the D-MSCs was demonstrated by the differentiation into osteogenic and adipogenic lineages for four weeks and stained with Alizarin red and Oil Red O, as previously described^41^.

### Stimulation of D-MSCs

D-MSCs populations were stimulated with 200 ng/ml neuregulin1-beta1 (R&D systems), 10ng/ml basic fibroblast growth factor (Millipore), 5ng/ml platelet derived growth factor (Millipore) and 14 µM forskolin (Sigma), for two weeks according to a protocol previously described for adipose stem cells^7^. Upon reaching 90% confluence, cells were passaged into new flasks. Cultures of unstimulated cells were run in parallel. Conditioned medium was collected from freshly stimulated cells after an initial two week expansion in the stimulating factors.

### Semi-quantitative and quantitative RT-PCR

RT-PCR was performed to measure mRNA expression levels of neurotrophic and angiogenic molecules. Total RNA was isolated from D-MSCs (passages 2–4) using an RNeasy™ kit (Qiagen) and then 1ng RNA was incorporated into the One-Step RT-PCR kit (Qiagen) per reaction mix. Reverse transcription and polymerase chain reactions were performed using primers from Sigma with parameters previously described^7^ and shown in Supplementary Table 1. For qRT-PCR, 10 ng/µl reaction mix was converted into cDNA using an iScript™ cDNA synthesis kit (Bio-Rad). qRT-PCR was performed using SsoFast™EvaGreen supermix (Bio-Rad) in a CFX96 Optical Cycler and analyzed using the CFX96 manager software (Bio-Rad). Reactions were optimized and processed according to the manufacturer with initial denaturation/DNA polymerase activation at 95 °C for 30 s followed by PCR: 95 °C for 5 s, variable annealing temperature (see Supplementary Table 1) for 5 s, and 65 °C for 5 s repeated for 40 cycles. Expression levels were normalized to 3 reference genes (*GAPDH, ACTB* and *RPL13A*). Data were calculated as relative expressions according to the ∆∆C(t) principle with SCAP samples set as value equal to 1.

### Enzyme-linked immunosorbent assay (ELISA)

Both stimulated and unstimulated cells were cultured in T25 flask (Nunc). A final volume of 8 ml of conditioned media was collected. The cell numbers were calculated (≈8 × 10^6^ ± 2 × 10^5^ cells/group). The conditioned media were analysed by ELISA using the ChemiKine™ BDNF sandwich ELISA kit (Millipore) or NGF, GDNF, angiopoietin-1 and VEGF-A sandwich ELISA kits (RayBiotech Inc) according to the manufacturer’s protocol and as previously described^7^. All samples were analysed in triplicate and the absorbance was measured at 450 nm on a SpectraMax190 microplate reader (Molecular Devices Inc). The quantity of factors (pg/ml) were calculated against standard curves produced using recombinant proteins provided in the kits and normalised to the final number of cells (1 ml conditioned media from 1 × 10^6^ cells). Intracellular production of one of the proteins, BDNF, was confirmed by immunostaining. In brief, after fixation and blocking with normal goat serum, rabbit anti-BDNF antibody (1:50, Santa Cruz Biotechnology) was applied to cell cultures for 2 h, followed by washing and application of secondary Alexa Fluor 568 conjugated goat anti-rabbit IgG (1:1000; Molecular Probes) for 1 h at room temperature in the dark. The slides were cover slipped with Prolong anti-fade mounting medium containing 4′-6-diamido-2-phenylindole (DAPI, Invitrogen).

### *In vitro* neurite outgrowth assay

Conditioned media (CM) from these flasks were collected at 48 hours after last medium change, centrifuged at 800 g for 10 minutes to remove any floating cells and then 0.5 ml of the supernatants were applied directly to 8-well Poly-D-Lysine coated culture slide (Corning) containing 400 retinoic acid-differentiated SHSY-5Y neuronal cells (ATCC), which had been plated 24 hours previously. Cultures were maintained for 48–72 hours before fixation with 4% (w/v) paraformaldehyde followed by immunostaining with βIII-tubulin antibody (1:500; Sigma) as described previously^49^. The resulting slides were analysed with an ECLIPSE 90i microscope and images captured with Nikon elements2 software (Nikon). The neurite outgrowths of the SH-SY5Y cells were manually traced with Image ProPlus software (MediaCybernetics). The percentage of cells with neurite outgrowth, total neurite length outgrowth, the longest neurite outgrowth, and neurite/cell were determined. To study the potential growth-promoting mechanisms of D-MSCs, neutralizing antibodies against BDNF (catalogue code: AB1513, Millipore) and VEGF-A (catalogue code: AF-293-NA, R&D Systems) were used. The antibodies were diluted 1:20 in conditioned media for 30 min at 20 °C, before the SH-SY5Y were exposed to the media.

### *In vivo* experiments

The experiments were performed on adult (10–12 weeks, n = 30, 5–8 rats per experimental group) female Sprague-Dawley rats (Charles River Laboratories, Germany). The animal care and experimental procedures were carried out in accordance with Directive 2010/63/EU of the European Parliament and of the Council on the protection of animals used for scientific purposes and also approved by the Northern Swedish Committee for Ethics in Animal Experiments (No. A182-12). All surgical procedures were performed under general anaesthesia using intraperitoneal injection of a mixture of ketamine (Ketalar®, Parke-Davis; 100 mg/kg i) and xylazine (Rompun®, Bayer; 10 mg/kg i.v.). After surgery, the rats were given the analgesic, Finadyne (Schering-Plough, Denmark; 2.5 mg/kg, subcutaneous; s.c.). Each animal was housed alone in a cage after surgery and exposed to 12-hour light/dark cycles, with free access to food and water.

Fibrin conduits were moulded from two-compound fibrin glue (Tisseel® Duo Quick, Baxter) to produce tubular 14mm long conduits^50^. Fibrin glue conduits were loaded with diluted fibrin matrix, with or without cells^9^. Either SCAP, DPSC, PDLSC or rat adult Schwann cells (isolated and maintained as previously described^51^) were seeded in the diluted fibrin matrix (2 × 10^6^/20 µl). Diluted thrombin solution (20 µl) was injected into the lumen of a conduit and the cells resuspended in dilute fibrinogen were added immediately.

Under an operating microscope (Carl Zeiss), the left sciatic nerve was exposed and transected 5mm below the sciatic notch. A 5 mm length of sciatic nerve was excised, to create a 10 mm gap. The transected nerve ends were inserted 2 mm into the fibrin conduits and the epineurium was fixed to the conduit with two 10.0 Ethilon sutures at each end. The muscle and skin was closed in layers. Animals were allowed to survive for 2 weeks and all the groups (including the empty conduit group) were treated daily with cyclosporine A (CsA; Sandimmun, Novartis) injected subcutaneously at 1.5 mg per 100 g body weight beginning 24 hours before surgery. Empty fibrin conduits (n = 5) served as baseline negative control.

### Tissue analyses

At the end of the survival period, the animals were killed with an overdose of sodium pentobarbital (240 mg/kg, intraperitoneal) and ipsilateral L4-L6 dorsal root ganglia (DRG) were harvested and rapidly frozen in liquid nitrogen for molecular analysis. The conduits were fixed for 24 h with 4% (w/v) paraformaldehyde, cryoprotected in 10%, 20% and 30% (w/v) sucrose, embedded in OCT™ medium and frozen at −85 °C. Serial longitudinal 16 µm thick sections were cut on a cryomicrotome (Leica Instruments, Germany), thaw-mounted onto SuperFrost®Plus slides, dried overnight at room temperature before immunostaining. After blocking with normal serum, mouse anti-βIII-tubulin antibody (1:500, Sigma-Aldrich), mouse anti-human nuclei antibody (1:100, Millipore), rabbit anti-S100 protein (1:2000, Dako), rabbit anti-BDNF (1:50, Santa Cruz Biotechnology) were applied for 2 h, followed by application of secondary Alexa Fluor 488 conjugated goat anti-mouse IgG or Alexa Fluor 568 conjugated goat anti-rabbit IgG (1:300; Molecular Probes) for 1 h at room temperature in the dark. The slides were coverslipped with Prolong anti-fade mounting medium containing 4′-6-diamido-2-phenylindole (DAPI, Invitrogen). Axon regeneration distance was measured using an optical microgrid from the beginning of the proximal nerve stump to the longest regenerating axon. qRT-PCR was performed (as described above) on the DRG. Primers and cycling conditions used were as previously described^7^.

### Statistical Analysis

For all statistical analyses in this study, one-way analysis of variance (ANOVA) followed by *post hoc* Newman–Keuls test (Prism®, Graph-Pad Software Inc) was used to determine statistical differences between experimental groups. Statistical significance was set as *p < 0.05, **p < 0.01, ***p < 0.001.

## Electronic supplementary material

Supplementary Information
