# Supplementary material for: The neurotrophic effects of different human dental mesenchymal stem cells
Source: Sci Rep. 2017 Oct 3;7:12605. doi: 10.1038/s41598-017-12969-1 (PMC5626751; doi:10.1038/s41598-017-12969-1)
Supplement: Supplementary file 1 — Supplementary Information [file 41598_2017_12969_MOESM1_ESM.doc]

**The neurotrophic effects of different human dental mesenchymal stem cells**

Mallappa K. Kolar^a,b,≠^, Vinay N. Itte^a,b,≠^, Paul J. Kingham^a^, Lev N. Novikov^a^, Mikael Wiberg^a,b^ and Peyman Kelk^a^*

≠ These authors contributed equally

a) Department of Integrative Medical Biology, Section for Anatomy, Umeå University, Umeå, Sweden

b) Department of Surgical & Perioperative Sciences, Section for Hand and Plastic Surgery, Umeå University, Umeå, Sweden


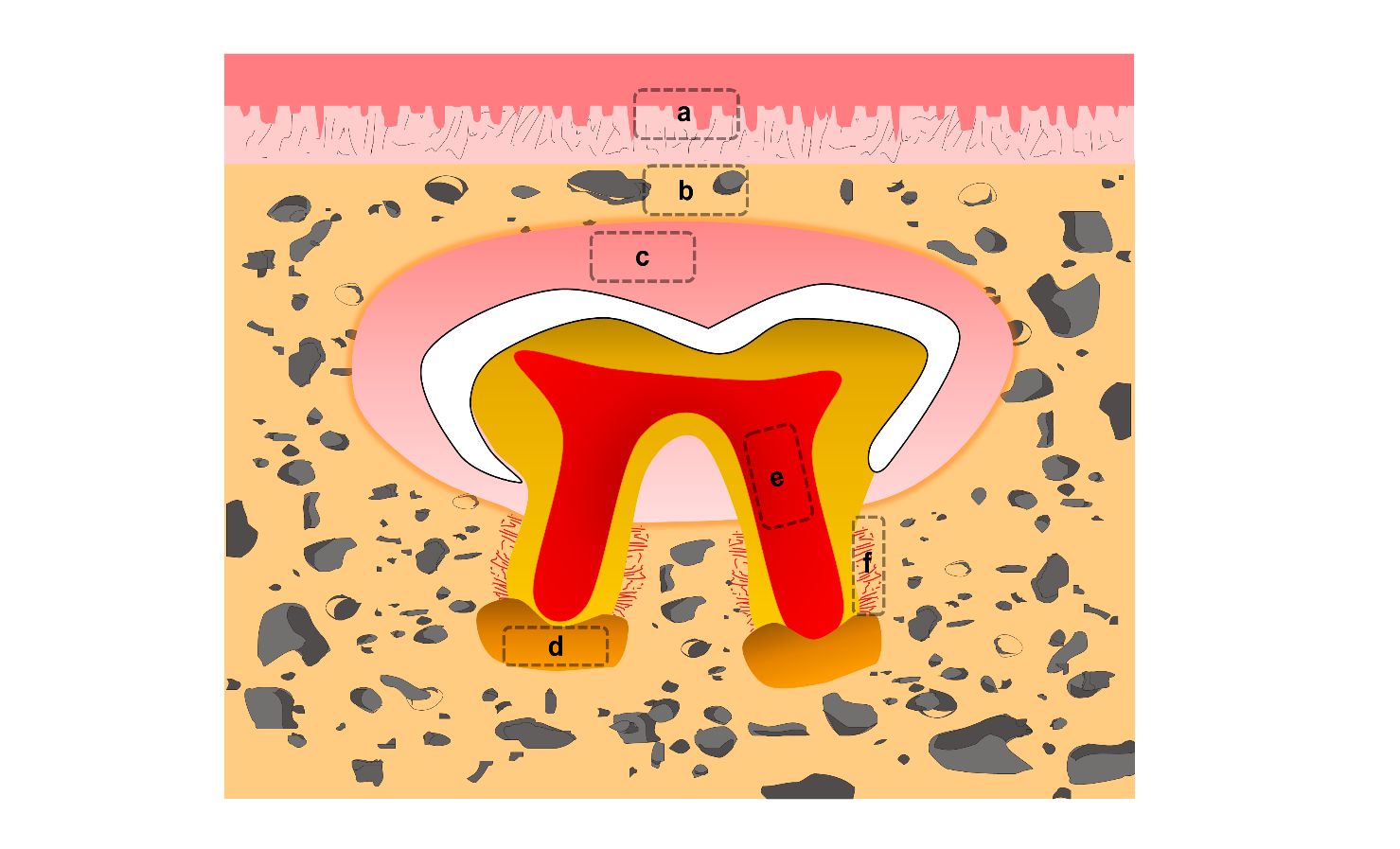


**Supplementary Figure 1.** Schematic illustration of the collection sites of Dental MSCs. Impacted premolars or third molars with approximately 30-70% root formations were selected and surgically removed by reflecting back the gum tissue (a) and removal of jaw-bone (b). Of the seven donors, two donors had impacted teeth with dental follicle (c) that only covered the “crown-part” of the teeth. In the surgically removed teeth from these two donors (with approximately 70% completed root formation), the various dental-MSCs could easily be distinguished: SCAP (d), DPSC (e), and PDLSC (f). This schematic illustration is original and was made by authors in Microsoft Powerpoint.


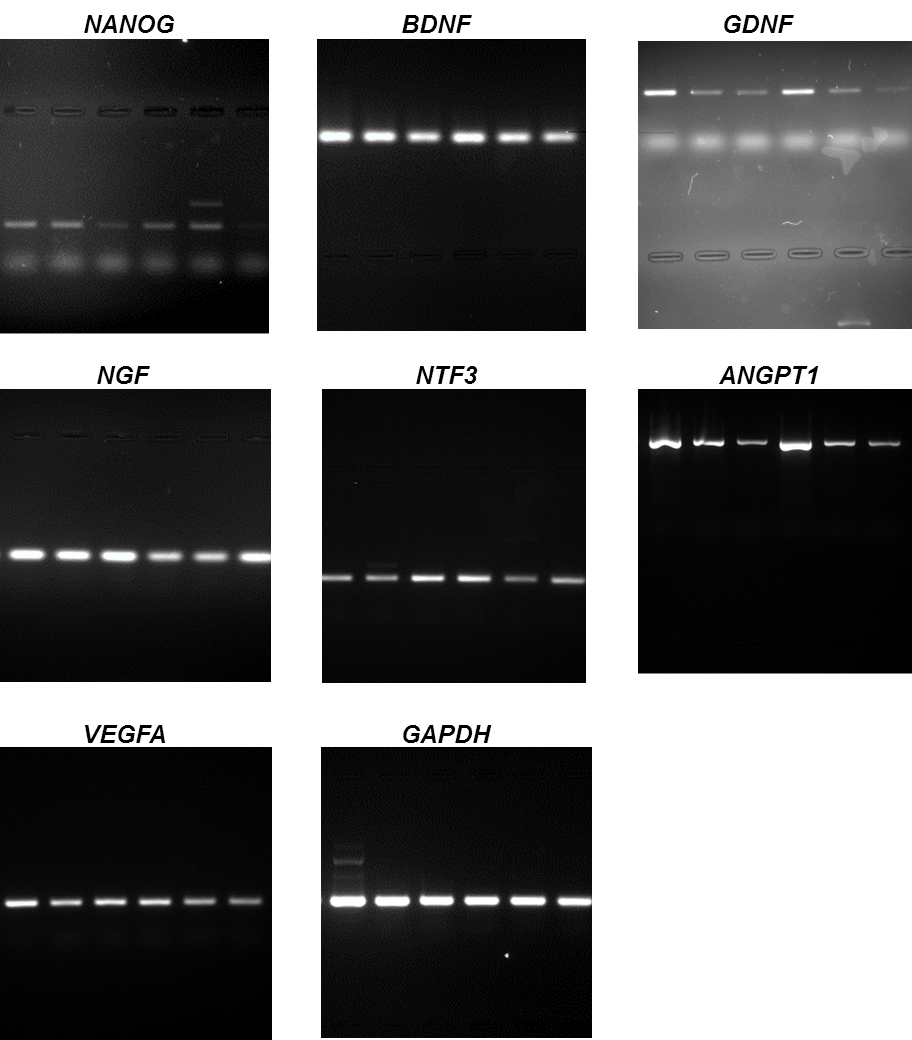


**Supplementary Figure 2.** Original full images of agarose gels shown in Figure 2. Neurotrophic and angiogenic factor gene expression of D-MSCs. RT-PCR analysis of various unstimulated D-MSCs at passage 2, showed a variability of gene expression in unstimulated D-MSCs. *GAPDH* is used as a house-keeping gene.


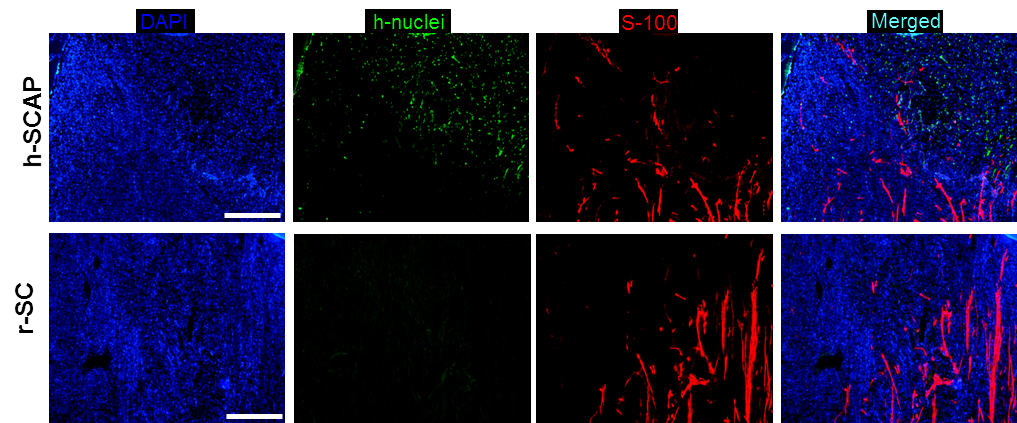


**Supplementary Figure 3.** Specificity of human-nuclei antibody. Sections from donor I SCAP and from rSC groups were stained with DAPI (blue), h-nuclei (green), and S100 (red). Human nuclei-specific antigen staining is only positive in the SCAP group. In addition, there was no co-localization of h-nuclei (green) with S100 antigen (red), indicating that the transplanted cells did not directly differentiate into Schwann cells. Scale bar: 250 µm.


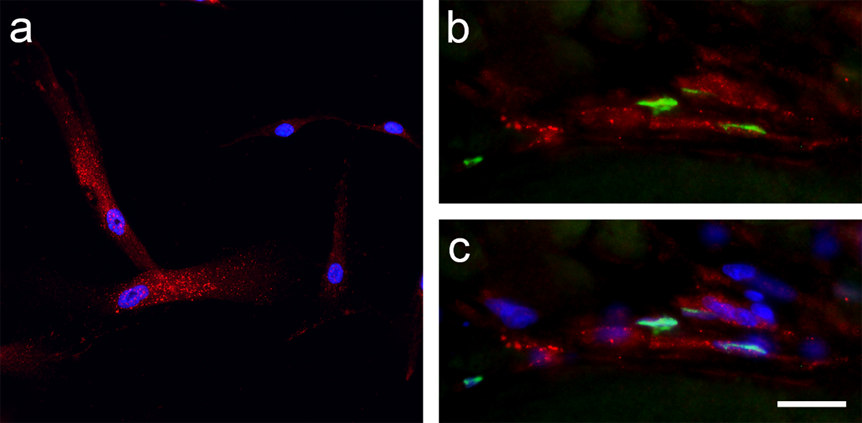


**Supplementary Figure 4.** Expression of BDNF in SCAP *in vitro* and *in vivo*. Unstimulated SCAP shown from donor I expressed BDNF *in vitro* (a). BDNF expression in the sciatic nerve injured rats after two weeks is illustrated in (b) and (c). The BDNF was found in the vicinity of the transplanted human cells as shown in (b), where the SCAP from donor I is stained with h-nuclei-specific antigen (green). DAPI (blue) staining represents all cells found in this section (c). Scale bar = 20 µm in (a) and 60 µm in (b) & (c).

***Gene Name Forward Sequence (5′→ 3′) Reverse Sequence (5′→ 3′) (°C)***

***NANOG*** CTCCTTCCATGGATCTGCTTATT GGTCTTCACCTGTTTGTAGCTGA 64.7

***BDNF*** AGAGGCTTGACATCATTGGCTG CAAAGGCACTTGACTACTGAGCATC 65.6

***GDNF*** CACCAGATAAACAAATGGCAGTGC CGACAGGTCATCATCAAAGGCG 66.0

***NGF*** ATACAGGCGGAACCACACTCAG GTCCACAGTAATGTTGCGGGTC 65.1

***NTF3*** GGGAGATCAAAACGGGCAAC ACAAGGCACACACACAGGAC 62.0

***ANGPT1^1^*** CTTGACCGTGAATCTGGAGC AGCAAGACATAACAGGTGAG 59.7

***ANGPT1^2^*** CAGAAAACAGTGGGAGAAGATATAACC TGCCATCGTGTTCTGGAAGA 63.1

***VEGFA*** TACCTCCACCATGCCAAGT TGCATTCACATTTGTTGTGC 61.2

***GAPDH*** GAAGGTGAAGGTCGGAGT CAAGCTTCCCGTTCTCAGC 59.6

***ACTB*** CGAGAAGATGACCCAGATCA CGTACAGGGATAGCACAGC 58.5

***RPL13A***  AAGTACCAGGCAGTGACAG CCTGTTTCCGTAGCCTCATG 58.0

**Supplementary Table 1.** Forward and Reverse Primer Pairs with Annealing Temperatures. ***^1^*** Semi-quantitative RT-PCR and ***^2^*** qRT-PCR
